# Mortality in COVID-19 older patients hospitalized in a geriatric ward: Is obesity protective?

**DOI:** 10.1186/s12877-023-03937-8

**Published:** 2023-04-11

**Authors:** Julien Lagrandeur, Pauline Putallaz, Hélène Krief, Christophe J. Büla, Martial Coutaz

**Affiliations:** 1 Service of Geriatric Medicine, Hospital of Valais, Avenue de La Fusion 27, 1920 Martigny, Switzerland; 2 Service of Geriatric Medicine and Geriatric Rehabilitation, Lausanne University Hospital and University of Lausanne, Mont-Paisible 16, 1011 Lausanne, Switzerland

**Keywords:** COVID-19, Mortality, Obesity, Older persons

## Abstract

**Backgrounds:**

To investigate the relationship between obesity and 30-day mortality in a cohort of older hospitalized COVID-19 inpatients.

**Methods:**

Included patients were aged 70 years or more; hospitalized in acute geriatric wards between March and December 2020; with a positive PCR for COVID-19; not candidate to intensive care unit admission. Clinical data were collected from patients electronic medical records. Data on 30-day mortality were retrieved from the hospital administrative database.

**Results:**

Patients included (*N* = 294) were on average 83.4 ± 6.7 years old, 50.7% were women, and 21.7% were obese (BMI > 30 kg/m^2^). At 30-day, 85 (28.9%) patients were deceased. Compared to survivors in bivariable analysis, deceased patients were older (84.6 ± 7.6 vs 83.0 ± 6.3 years), more frequently with very complex health status (63.5% vs 39.7%, *P* < .001), but less frequently obese (13.4% vs 24.9%, *P* = .033) at admission. Over their stay, deceased patients more frequently (all *P* < .001) developed radiologic signs of COVID-19 (84.7% vs 58.9%), anorexia (84.7% vs 59.8%), hypernatremia (40.0% vs 10.5%), delirium (74.1% vs 30.1%), and need for oxygen (87.1% vs 46.4%) compared to survivors.

In multivariable analysis that controlled for all markers of poor prognosis identified in bivariable analysis, obese patients remain with 64% (adjOR 0.36, 95%CI 0.14–0.95, *P* = .038) lower odds to be deceased at 30-day than non-obese patients.

**Conclusions:**

In this population of older COVID-19 inpatients, an inverse association between obesity and 30-day mortality was observed even after adjusting for all already-known markers of poor prognosis. This result challenges previous observations in younger cohorts and would need to be replicated.

**Supplementary Information:**

The online version contains supplementary material available at 10.1186/s12877-023-03937-8.

## Backgrounds

Obesity, defined as a Body Mass Index (BMI) of 30.0 kg/m^2^ or greater, has been identified early in the pandemic as a risk factor for severe illness and death from SARS-CoV-2 infection in adults [1–6].

However, several later studies observed that the association between obesity and death was most pronounced among patients younger than 65 years and became weaker or even disappeared in older COVID-19 patients [6, 7].

Another study that observed a significant overall association between BMI and an increased mortality reported that this association remained only for extreme BMI (i.e., > 45 kg/m^2^) when restricting the analysis to patients aged over 60 years [8]. Moreover, a study restricted to a cohort of 290 hospitalized older persons aged 65 years or more (mean age 77.6 years) did not find any significant association between an increased BMI (30.7% of participants with BMI above 30 kg/m^2^) and COVID-19 mortality [9]. Accordingly, although some recently developed scores to predict in-hospital COVID-19 mortality includes obesity, [10] others do not [11, 12].

The relationship between health trajectories, all-cause mortality and obesity in old and very old persons has indeed been a subject of controversy. Although a “U-shaped” curve was observed in most studies among persons aged 65 to 74 years, with a significant increased mortality associated with high BMI (> 30 kg/m^2^), this relationship was weaker, absent, or even reversed in some analyses restricted to individuals aged over 75 or 80 years [13–17].

The present study was undertaken to get further insight on the relationship between obesity and COVID-19 mortality in a cohort of older patients aged 70 years or more hospitalized during the first and second pandemic waves in Switzerland. The hypothesis was that obese older patients hospitalized with COVID-19 will have similar mortality than older patients with normal weight because the proinflammatory state associated with obesity might be less pronounced in older age and possibly counterbalanced by their nutritional reserve. An additional aim was to investigate whether this relationship would vary according to the presence of anorexia, the most prevalent gastrointestinal symptoms that has been found to affect up to a quarter of COVID-19 patients [18].

## Methods

### Setting and study population

This retrospective study was conducted at a 900-bed reference hospital. Patients included in this analysis were those aged 70 years or more; hospitalized in the service of geriatric medicine between March 24 and December 31, 2020 with a diagnosis of COVID-19 confirmed by PCR; with advanced care directives or proxies directives declining transfer to an intensive care unit (ICU) in case of deterioration.

Patients were managed according to evolving standard of care and COVID-19 guidelines that were applied at the time of the study period. In particular, patients hospitalized during COVID-19 s wave (October to December 2020) who required oxygen support also benefited from systematic corticosteroid therapy (dexamethasone 4 to 6 mg/day up to 10 days) [19]. Management included oxygen support (but no endo-tracheal intubation or high flow ventilation), enteral and parenteral hydration support, thromboembolic prevention, and antibiotic treatments in case of superinfection. In addition, physical therapy and dietitian support were offered, including nutritional and vitamin supplements (vitamin B12, folates, vitamin D) when needed. No antiviral drugs or monoclonal antibody treatments were offered at that time.

### Characteristics collected at admission

At hospital admission, information was collected on patients’ socio-demographics (age, gender), functional (ADL, IADL) and cognitive status (MMS) [20–22], as well as the presence of the following comorbid conditions that have been previously associated with COVID-19 unfavorable outcome: type 2 diabetes, hypertension, cardiovascular disease (congestive heart failure, coronary heart disease, etc.), chronic respiratory disease.

Based on their cognitive and functional status, comorbidities, and estimated remaining life expectancy assessed according to Swiss life time table and clinically, by one senior geriatrician, patients were further classified according to the American Diabetes Association (ADA) as healthy, with complex, or very complex health profile [23].

### Body Mass Index (BMI) assessment

BMI was assessed according to self-reported height and measured weight upon hospital admission. Obesity was defined as a BMI ≥ 30.0 kg/m2, overweight as a BMI between 25.0 to 29.9 kg/m^2^, normal weight as a BMI between 18.5–24.9 kg/m^2^, and underweight as a BMI < 18.5 kg/m^2^. BMI could not be calculated in 3 patients due to missing weight and/or height [24].

### Data collected over the hospital stay

Over the hospital stay, information was collected on the apparition of the following symptom and signs: anorexia (defined as a caloric intake < 50% of estimated daily requirement over at least three consecutive days), desaturation with oxygen requirement (defined as an O2 saturation < 92% at room air), delirium (according to CAM-based clinical criteria), radiologic signs of COVID-19 pneumonia (uni- or bilateral infiltrate at chest x-ray or CT-scan), hypernatremia (> 145 mmol/L), C-reactive protein level (≥ 50 mg/L, maximum level over the stay), Procalcitonin level (≥ 0.25 ng/ml, maximum level over the stay).

### 30-day mortality

Mortality at 30-day follow-up was retrieved from the hospital database for patients who were still hospitalized. For discharged patients, information on mortality was collected through in-person contact with the patient, a proxy/relative, and/or the family physician to ascertain living status.

### Statistical analysis

Simple usual statistics (mean, proportions) were used to describe the characteristics of the study population. Characteristics of patients who were deceased or not at 30-day follow-up were compared in bivariable analysis by parametric (Student’s t test and Chi-square tests) and non-parametric (Kruskall-Wallis and Fisher exact tests) tests, according to distribution. Characteristics significantly (*P* < 0.050) associated with 30-day mortality in bivariable analysis were considered as candidate in a multivariable logistic regression model. Results for the variables included in the final model are presented as adjusted odds ratios (adjOR) with 95% confidence intervals (95%CI). The area under the ROC curve (AUROC) was calculated (c-statistic).

To further investigate the association between obesity, anorexia, and 30-day mortality, the proportion of deceased patients among those obese (BMI ≥ 30.0 kg/m^2^) or not (BMI < 30.0 kg/m^2^), and those with or without anorexia were computed and compared, using Pearson Chi-square test and Fisher’s exact test according to distribution of patients. All analyses were performed on Stata software, version 16.

### Ethical approval

The study was approved by the Canton of Valais ethics Committee (Commission Cantonale d’éthique médicale, CCVEM) and was granted an exemption from requiring individual written.informed consent. (decision of January 22th, 2021).

## Results

Over the study period, a total of 294 admitted patients fulfilled the eligibility criteria (Table 1). Population mean age was 83.4 ± 6.7 years and 50.7% were women, mostly living at home (80.8%) whereas only 7.3% were admitted from a long term care facility. Almost half (46.6%) had a very complex health profile by ADA criteria, and more than three quarters were diagnosed with hypertension (77.9%) and cardiovascular diseases (79.6%). Cognitive impairment was present in about half (53.7%).

**Table 1:** Characteristics of the study population and results of their comparisons in patients deceased or not by 30-day follow-up

	Total population	Deceased by 30-day follow-up		*P* value*
		Yes	No	
	(*N* = 294) (100.0%)	(*N* = 85) (28.9%)	(*N* = 209) (71.1%)	
**Characteristics at admission**				
Age, mean [± SD]	83.4 [± 6.7]	84.6 [± 7.6]	83.0 [± 6.3]	.059
Women, n (%)	149 (50.7)	37 (43.5)	112 (53.6)	.118
Health profile according to ADA^a^, n (%)				
• Very complex	137 (46.6)	54 (63.5)	83 (39.7)	
• Complex or robust	157 (53.4)	31 (36.5)	126 (60.3)	< .001
Cardiovascular diseases, n (%)	234 (79.6)	68 (80.0)	166 (79.4)	.912
Hypertension, n (%)	229 (77.9)	68 (80.0)	161 (77.0)	.578
Cognitive impairment, n (%)	158 (53.7)	51 (60.0)	107 (51.2)	.170
Chronic respiratory disease, n (%)	73 (24.8)	25 (29.4)	48 (23.0)	.246
Diabetes, n (%)	70 (23.9)	14 (16.5)	56 (26.9)	.057
Obesity^b^, n (%)	63 (21.7)	11 (13.4)	52 (24.9)	.033
**Data from hospital stay**				
Abnormal chest X-ray, n (%)	195 (66.3)	72 (84.7)	123 (58.9)	< .001
Anorexia, n (%)	197 (67.0)	72 (84.7)	125 (59.8)	< .001
Delirium, n (%)	126 (42.9)	63 (74.1)	63 (30.1)	< .001
Hypernatremia, n (%)	56 (19.1)	34 (40.0)	22 (10.5)	< .001
Procalcitonine positive^c^n (%)	119 (40.5)	52 (61.2)	19 (9.1)	< .001
CRP positive^d^n (%)	220 (74.8)	77 (90.6)	143 (68.5)	< .001
Oxygen therapy, n (%)	171 (58.2)	74 (87.1)	97 (46.4)	< .001

^*^From Student’s t test and Chi-square test for continuous and categorical variables, respectively

^a^As defined by the American Diabetes Association (ADA) [20]

^b^Defined as a BMI ≥ 30.0 kg/m^2^; *N* = 3 missing BMI

^c^Defined as a Procalcitonin level ≥ 0.25 ng/mL

^d^Defined as a CRP level ≥ 50 mg/L

Overall, about a fifth (21.7%) of patients fulfilled criteria for obesity.

### Characteristics associated with 30-day mortality (Table 1)

**Fig. 1:**
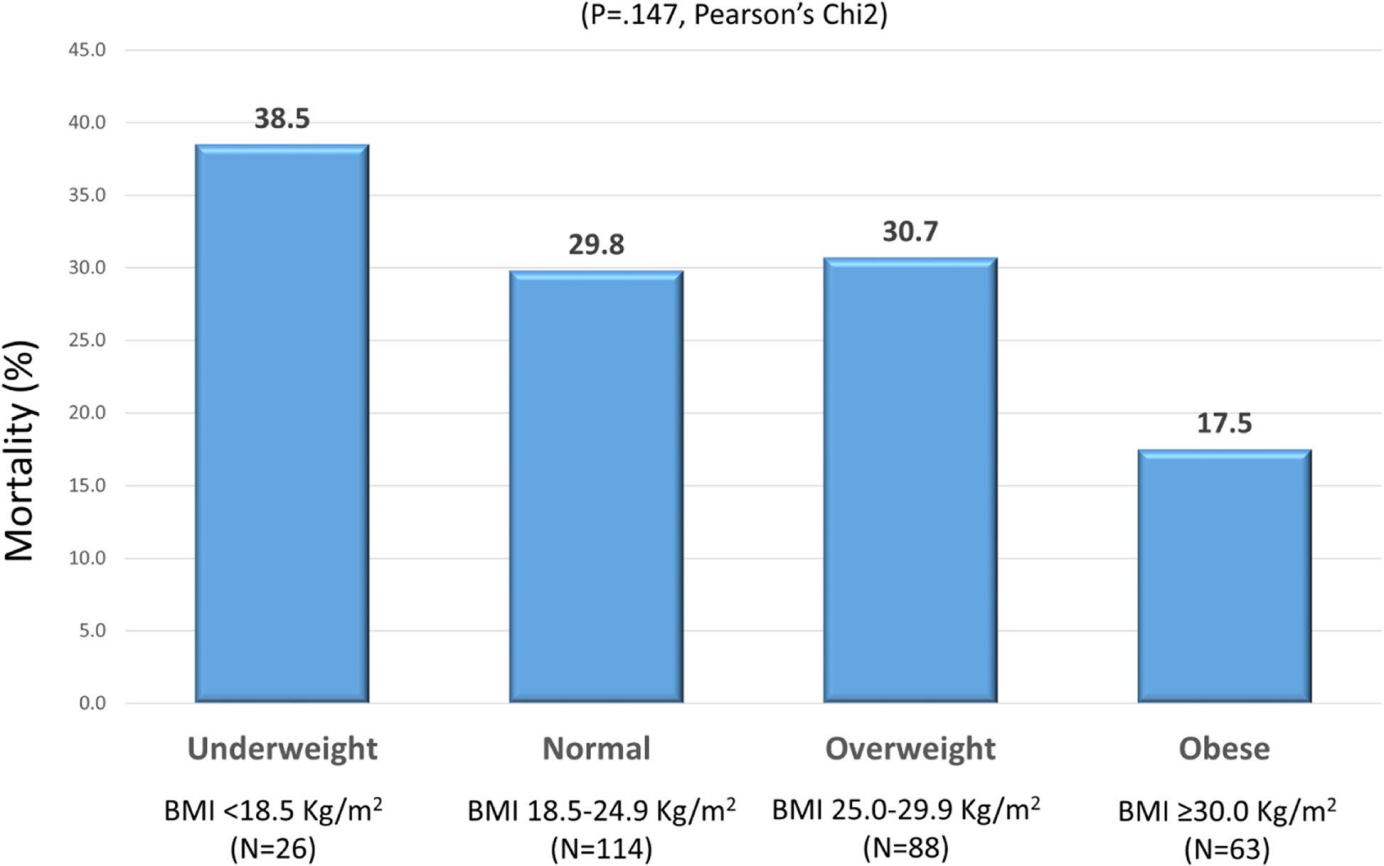
Proportions of deceased patients according to Body Mass Index (BMI) categories. (*N* = 3 missing BMI). OB + : Obese patients (BMI > 30 kg/m^2^). OB -: Non obese patients (BMI < 30 kg/^2^). PCT + : Procalcitonine maximum level ≥ 0.25 ng/ml. PCT -: Procalcitonine maximum level < 0.25 ng/mL

Among patients’ characteristics present at admission, a very complex health profile was more frequent among deceased than surviving patients (63.5% vs 39.7%, *P* < 0.001) whereas obesity was significantly less prevalent in deceased than surviving patients (13.4% vs 24.9%, *P* = 0.033). When further stratifying mortality by nutritional status, a trend toward progressively lower mortality was apparent from underweighted to obese patients (Fig. 1).

Patients with radiologic signs (at admission or subsequently) of COVID-19 at chest X-ray or CT scan, and those who developed over their hospital stay anorexia, delirium, hypernatremia, the need for oxygen therapy, or high level of inflammatory markers (Protein-C, Procalcitonin) were significantly more likely to be deceased by 30-day (all *P* < 0.001).

**Fig. 2:**
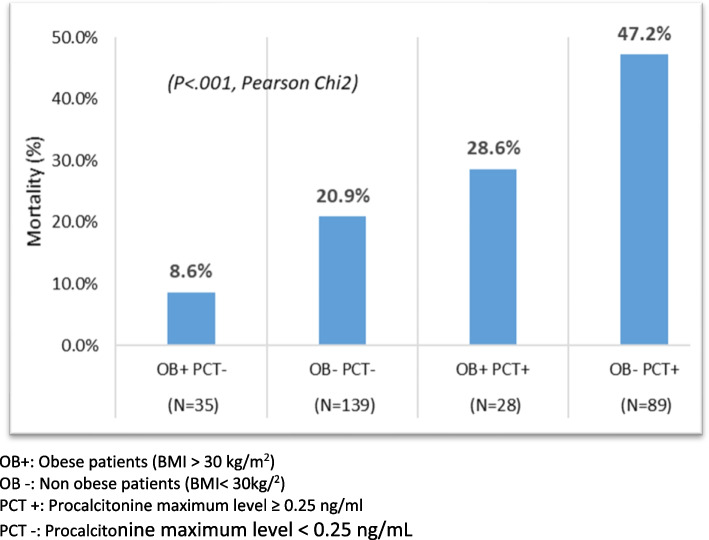
Proportions of deceased patients according to the presence ( +) or absence (-) of obesity (OB) and PCT status (*N* = 3 missing BMI)

Figure 2 further illustrates the interaction between obesity and inflammatory markers as procalcitonin in showing a steadily incremental mortality from obese patients with negative inflammatory markers (8.6% mortality) to non-obese patients with inflammation (47.5% mortality).

**Fig. 3:**
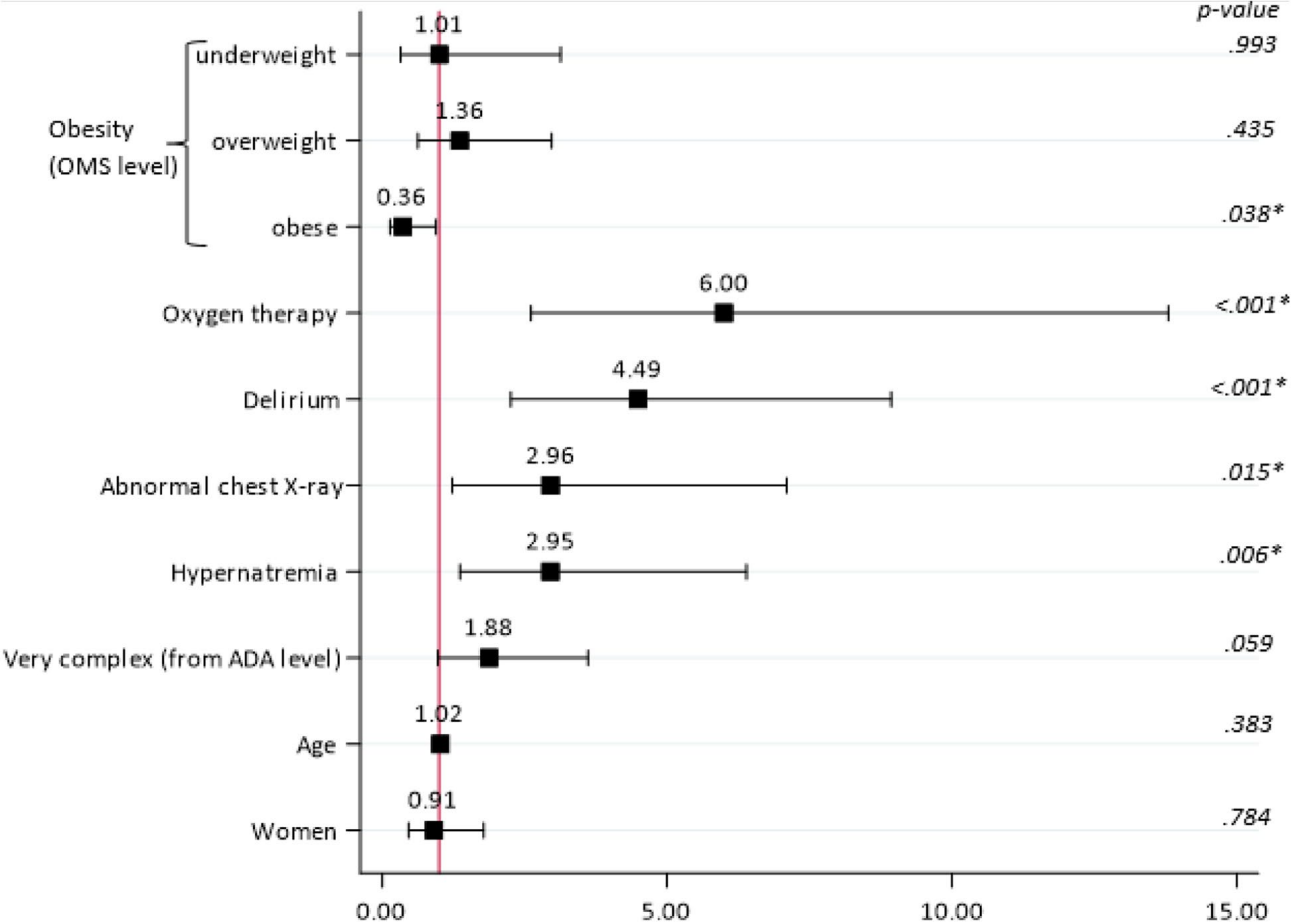
Results from multivariable logistic regression analysis with corresponding adjusted odds ratios and 95% confidence intervals

A similar interaction was also observed between Fig. 3 (Additional file 1: Appendix) illustrate the interaction between obesity and anorexia (Additional file 1: Supplementary Figure 1) with a steadily incremental mortality from obese patient without anorexia (no death) to non obese patient with anorexia (39.5% mortality)**.**

In multivariable analysis (Fig. 3) that adjusted for age and sex in addition to covariates that were significant in bivariable analysis, obese patients remained with 64% (adjOR 0.36, 95%CI 0.14–0.95, *P* = 0.038) lower odds to be deceased at 30-day than non-obese patients.

Inversely, patients who, over their stay, required oxygen therapy (adjOR 6.00, 95%CI 2.61–13.80, *P* < 0.001), developed a delirium (adjOR 4.49, 95%CI 2.26–8.93, *P* < 0.001), showed radiologic signs of COVID-19 (adjOR 2.96, 95%CI 1.23–7.10, *P* = 0.015), developed a hypernatremia (adjOR 2.95, 95%CI 1.37–6.39, *P* = 0.006) remained at increased probability of being deceased at 30-day follow-up. In addition, patients with a very complex dependency profile at admission also tended to be at increased risk of 30-day mortality (adjOR 1.88, 95%CI 0.98–3.61, *P* = 0.059). Anorexia, CRP, and procalcitonin level did not remain independently associated with mortality and no significant interaction with obesity was observed, thus it was removed from the final model.

This multivariable model showed good accuracy with an area under the ROC curve (AUROC) of 86.6%, a sensitivity of 57.3% and a specificity of 89.0%.

## Discussion

This study investigates the relationship between obesity and COVID-19 mortality at 30-day in a cohort of older patients who were not candidate for an ICU admission. Results show that, even when controlling for several well-known markers of COVID-19 severity and poor prognosis, obese patients remained at about 60% lower odds to be deceased at 30-day follow-up.

These results are important because they challenge earlier observations that reported an increased mortality among obese COVID-19 patients, independent of age [2–4]. Although several previous studies in older population questioned this association by observing its disappearance in older cohorts, [6, 7, 9] the present study is the first, to our knowledge, to report a significant independent inverse association between obesity and COVID-19 mortality. Indeed, a recent study in a similar cohort of hospitalized octogenarians also reported a protective effect of higher subcutaneous fat area against mortality [25]. Several hypotheses can be proposed to explain the discordant observations. Most importantly, the age range of the present cohort is notably older than in most cohorts from earlier studies [1–6].

As observed in other studies [26], patients from the most impaired group, according to the proxy measure of frailty used in the current study, had almost twice higher odds of dying. However, this association did not remain significant after adjustment for factors reflecting disease severity such as O2 requirement or delirium.

A noteworthy difference with previous studies is indeed our ability to adjust for a large set of robust markers of COVID-19 infection’s severity and risk of mortality, a clear strength of the present report. Likely, obesity was protective in this cohort of mostly frail older patients with very complex health profile because it allowed these patients to overcome the weight loss associated with COVID-19 [25].

This is further suggested by results of the analysis that investigated the combined effect of obesity and anorexia. Although no significant interaction was observed in our study, this analysis was unfortunately hampered by the absence of any death among obese patients without anorexia. Future studies with larger population should further examine this interaction, but anorexia should still be considered as a clinically useful marker of reserved prognosis in similar patients.

Finally, an alternative hypothesis could be that a selection bias related to the exclusion of COVID-19 patients who were candidates to intensive care occurred. Indeed, only two-thirds of the cohort had signs of pulmonary SARS-CoV-2 involvement, a proportion that is lower than reported in other studies. Patients in this cohort might thus less prone to suffer from the detrimental mechanical and pro-inflammatory effects of obesity [27–29].

Other results from the multivariable analysis are very consistent with those of previous studies that investigated risk factors for COVID-19 mortality in older patients. This observation further support the robustness of our main finding about the protective role of obesity in this study population.

This study has several limitations. First, BMI was calculated from self-reported rather than measured height. The WHO definition for BMI cut-offs could be adapted to this specific old population, but the levels are debated in literature. Second, the study was performed in a single-center and generalization to other health care environment should be cautious. Third, a formal measure of frailty and sarcopenia was not performed routinely, even though the ADA classification in healthy, complex, and very complex could be considered as a proxy measure. Finally, the small size of some subgroup limited our ability to perform some analysis such as about the interaction between obesity and anorexia.

This study has however several strengths such the large set of covariates that allowed to adjusting for most relevant comorbidities and risk factors previously associated with COVID-19 mortality, and the complete follow-up for the mortality outcome assessment.

## Conclusions

We found that obesity was strongly and independently associated with a reduced risk of death in this cohort of older patients hospitalized in geriatric wards. Results also confirmed the already-known associations between dependency before admission, as well as signs of unfavorable evolution after admission with COVID-19 mortality. The independent inverse association between obesity and 30-day mortality challenges previous observations in younger cohorts, but this finding would need to be further studied.

## Supplementary Information

**Additional file 1: Figure S1.** Proportions of deceased patients accordingto the presence (+) orabsence (-) of obesity (OB) and anorexia (ANO)(N=3 missing BMI).

**Additional file 2: Table S1.**

## Data Availability

The anonymized datasets used and analysed during the current study are available in the submission’s additional supporting files.

## Abbreviations

PCR: Polymerase chain reaction
BMI: Body mass index
ICU: Intensive care unit
ADL: Activity of daily living
IADL: Instrumental activity of daily living
MMS: Mini mental stateADA: American diabete association
CT scan: Computed tomography scanner
AdjOR: Adjust odd ratio
CI: Confidence interval
MMSE: Mini mental status exam
