# Supplementary material for: Mortality in COVID-19 older patients hospitalized in a geriatric ward: Is obesity protective?
Source: BMC Geriatr. 2023 Apr 11;23:228. doi: 10.1186/s12877-023-03937-8 (PMC10088129; doi:10.1186/s12877-023-03937-8)

**Appendix**

**Supplementary Figure 1.** Proportions of deceased patients according to the presence (+) or absence (-) of obesity (OB) and anorexia (ANO) (N=3 missing BMI).


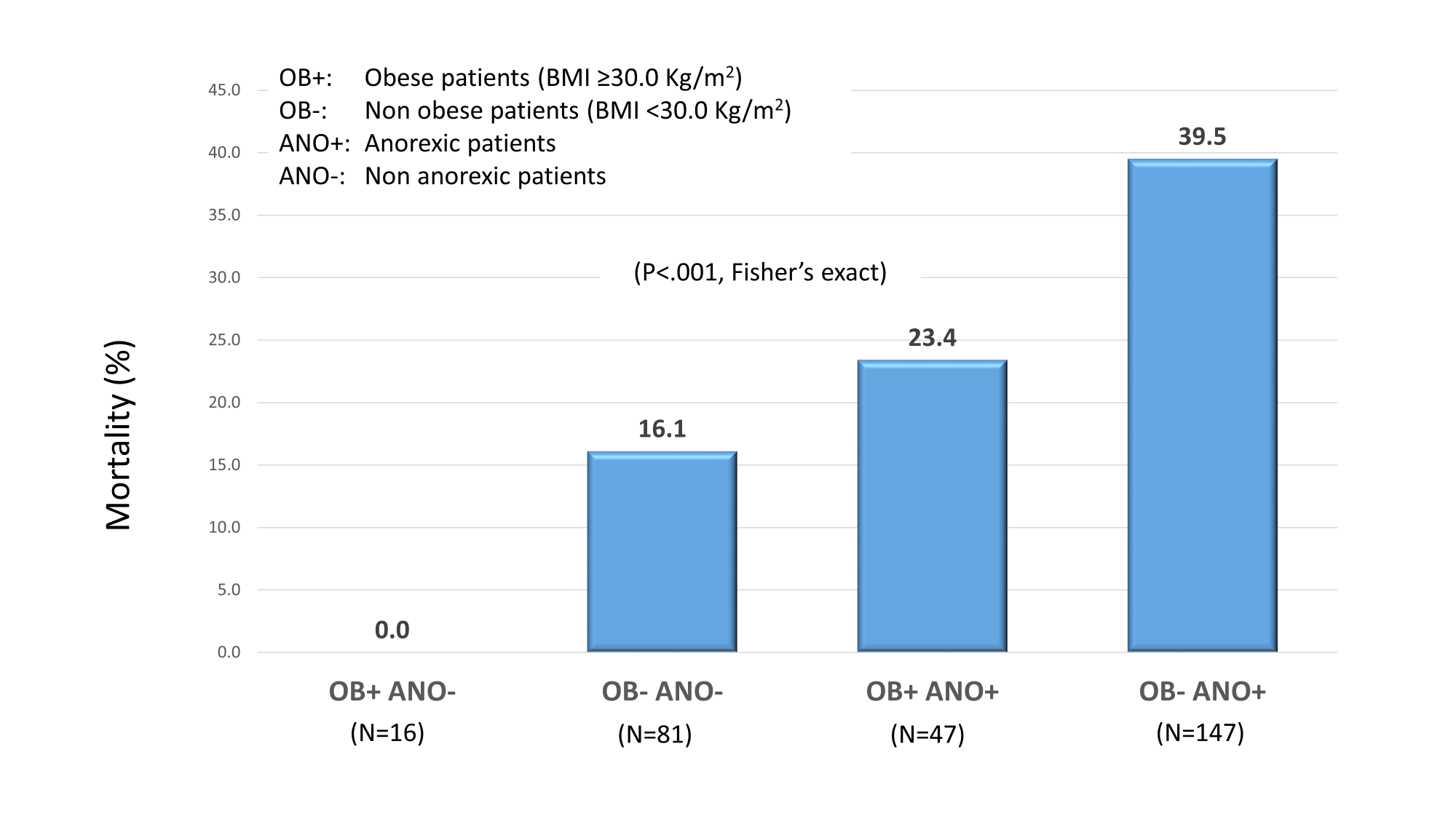

Supplement: Supplementary file 1 — Additional file 1: Figure S1. Proportions of deceased patients accordingto the presence (+) orabsence (-) of obesity (OB) and anorexia (ANO)(N=3 missing BMI). [file 12877_2023_3937_MOESM1_ESM.docx]
